# Applicability of Motivational Interviewing for Chronic Disease Management in Primary Care Following a Web-Based E-Learning Course: Cross-Sectional Study

**DOI:** 10.2196/12540

**Published:** 2019-04-29

**Authors:** Karoline Lukaschek, Nico Schneider, Mercedes Schelle, Ulrik Bak Kirk, Tina Eriksson, Ilkka Kunnamo, Andrée Rochfort, Claire Collins, Jochen Gensichen

**Affiliations:** 1 Institute of General Practice and Family Medicine University Hospital Ludwig-Maximilians-Universität München Munich Germany; 2 Institute of Psychosocial Medicine and Psychotherapy Jena University Hospital University of Jena Jena Germany; 3 Institute of General Practice and Family Medicine Jena University Hospital University of Jena Jena Germany; 4 European Society for Quality and Safety in Family Practice København Denmark; 5 The Finnish Medical Society Duodecim Helsinki Finland; 6 Irish College of General Practitioners Dublin Ireland

**Keywords:** motivational interviewing, e-learning, web-based learning, primary care, health behavior change, disease management

## Abstract

**Background:**

Motivational interviewing (MI) is an established communication method for enhancing intrinsic motivation for changing health behavior. E-learning can reduce the cost and time involved in providing continuing education and can be easily integrated into individual working arrangements and the daily routines of medical professionals. Thus, a Web-based course was devised to familiarize health professionals with different levels of education and expertise with MI techniques for patients with chronic conditions.

**Objective:**

The aim of this study was to report participants’ opinion on the practicality of MI (as learned in the course) in daily practice, stratified by the level of education.

**Methods:**

Participants (N=607) of the MI Web-based training course evaluated the course over 18 months, using a self-administered questionnaire. The evaluation was analyzed descriptively and stratified for the level of education (medical students, physicians in specialist training [PSTs], and general practitioners [GPs]).

**Results:**

Participants rated the applicability of the skills and knowledge gained by the course as positive (medical students: 94% [79/84] *good*; PSTs: 88.6% [109/123] *excellent*; and GPs: 51.3% [182/355] *excellent*). When asked whether they envisage the use of MI in the future, 79% (67/84) of the students stated *to a certain extent*, 88.6% (109/123) of the PSTs stated to *a great extent*, and 38.6% (137/355) of GPs stated *to a great extent*. Participants acknowledged an improvement of communication skills such as inviting (medical students: 85% [72/84]; PSTs: 90.2% [111/123]; GPs: 37.2% [132/355]) and encouraging (medical students: 81% [68/84]; PSTs: 45.5% [56/123]; GPs: 36.3% [129/355]) patients to talk about behavior change and conveying respect for patient’s choices (medical students: 72% [61/84]; PSTs: 50.0% [61/123]; GPs: 23.4% [83/355]).

**Conclusions:**

Participants confirmed the practicality of MI. However, the extent to which the practicality of MI was acknowledged as well as its expected benefits depended on the individual’s level of education/expertise.

## Introduction

### Background

Motivational interviewing (MI) is an evidence-based client-centered method of intervention focused on enhancing intrinsic motivation and behavior change by exploring and resolving ambivalence [1,2]. The term *motivational interviewing* was first coined by US psychologist Professor William Miller in 1983 and evolved from his experiences supporting problem drinkers [3]. It has since been applied to a wide range of behavior change, including weight loss, exercise, managing long-term conditions, pain management, anxiety and depression, and other issues where behavior change and self-confidence are desired.

A large body of literature studying the effectiveness of MI has led to several systematic reviews and meta-analyses published in support of MI effectiveness for a range of client outcomes and settings, including primary care [2,4-10]. Training professionals in MI not only improves their skills but also seems to have follow-on impacts for patients [11]. Although MI can improve the doctor-patient relationship and efficiency of the consultation [12], general practitioners (GPs) often lack time, basic training, or continuous education opportunities to update their knowledge and skills regarding clients’ motivation for change [6,13].

E-learning, defined as instruction delivered on a digital device [14], can reduce the cost and time involved in providing continuing education, as it is decentralized and asynchronous [15,16]. It offers flexibility with regard to learning times and locations and thus can be easily integrated in individual working arrangements and the daily routines of medical professionals [17]. Knowledge acquisition and clinical skill development by e-learning have been shown to be equal or superior to those observed with face-to-face instruction [18,19].

### Objectives

Given the already extensive literature on the effectiveness of MI for a range of client populations and of e-learning for medical professions, we focused on the individuals participating in the Web-based MI training course. Following the Dreyfus and Dreyfus model, each developmental stage of skill acquisition, moving from novice to expert, is characterized by differences in knowledge, skills, attitudes, and performance [20]. Thus, this study primarily aimed to investigate how physicians with different clinical experience levels (medical students, physicians in further education in general medicine, and specialists in general medicine), who had participated in an online training course on MI, evaluated the applicability of MI in their clinical practice. This study also aimed to assess if the Web-based MI training course would have an effect on their intention to use MI and their self-reported communication skills during MI.

## Methods

### Study Design

We conducted descriptive research using a cross-sectional study design to investigate the acceptability of a Web-based training course on MI for physicians with different clinical experience levels. The ethics committee of the Friedrich Schiller University (Jena, Germany), Medical Faculty, approved the study on June 18, 2014 (approval number 4120-0614).

### Participants

Participation in both the course and the subsequent survey was open to GPs (physicians with a completed medical specialist training, *Facharzt*), physicians in specialist training (PSTs; postgraduate clinical specialization training), medical students (undergraduate medical training), medical employees (eg, paramedics), and (examined) nurses. Professions were self-reported.

The project was advertised in German and English language medical journals specialized in general practice, at medical congresses (eg, Deutschen Gesellschaft für Allgemeinmedizin und Familienmedizin, World Organization of National Colleges, Academies and Academic Associations of General Practitioners/Family Physicians Europe, European Academy of Teachers in General Practice), and through the specialist distribution lists of general medicine departments or general medical professional associations.

### Motivational Interviewing E-Learning Course

#### Course Developers

The European Society for Quality and Safety in Family Practice (EQuiP) [21] developed a Web-based training course on MI consultation for family physicians regarding obesity and alcohol abuse, chronic conditions frequently encountered in primary care.

#### Development Process

The course followed the model developed by the Finnish Medical Society Duodecim. The international author team culturally adapted the course to the German and English language setting.

#### Revision and Updating

The course and its contents could not be changed during the observation period.

#### Quality Assurance

The course was evaluated using an online questionnaire (see below). No further quality assurance was performed.

#### Digital Preservation

The course and evaluation were only available for the study period.

**Table 1:** Questions and answers of the evaluation of the Web-based course on motivational interviewing.

Item	Question	Answers
1	My profession	Medical student/general practitioner/physician in specialist training/nurse/other
2	I took the course	In my workplace between other jobs/at home in my own time/at a school or university in association with my studies
3	Applicability of the skills and knowledge I obtained from the course in my clinical practice	Excellent/good/fair/poor
4	I feel that the course helped to improve my skills and competence	Fully agree/partly agree/do not agree
5	The course motivated me to learn and reflect upon the topic	Yes/somewhat/only a little/not at all
6	The following teaching methods are particularly useful in supporting my learning	Text parts/photos, drawn images/videos/speech, sound tracks/interactive animations/the examination
7	After taking the course I know better how to invite the patient to talk about behavior change.	Not at all/minimal/to some extent/a certain extent/a great extent
8	After taking the course I know better how to encourage the patient to talk about behavior change.	Not at all/minimal/to some extent/a certain extent/a great extent
9	After taking the course I know better how to actively convey respect for the patient’s choice about behavior change.	Not at all/minimal/to some extent/a certain extent/a great extent
10	After taking the course I and the patient exchange ideas about how the patient could change current behavior.	Not at all/minimal/to some extent/a certain extent/a great extent
11	In MI^a^ it is important to listen empathically to the problems, but not engaging in them, to really concentrate and act on change talk. After taking the course I know better how to get a good balance in my consultations between empathic listening to problems and active engagement in change talk.	Not at all/minimal/to some extent/a certain extent/a great extent
12	In MI it is important to know that the tool is not useful for every consultation. After taking the course I know better how to select the right patients at the right time to use MI.	Not at all/minimal/to some extent/a certain extent/a great extent
13	After taking the course I think it is feasible for me to perform MI (with some patients) in my daily general practice.	Not at all/minimal/to some extent/a certain extent/a great extent
14	After taking the course I plan to use MI in my daily practice.	Not at all/minimal/to some extent/a certain extent/a great extent
15	The most beneficial aspect of the course for me was	Free text
16	My suggestions to improve the course	Free text
17	I would like to have e-learning courses of the following topics in the future	Free text

^a^MI: motivational interviewing.

#### Access

The course was available in an English and a German version. The English language course is available online [22]. The voluntary evaluation in the English or German language [23] started directly after completing the course. Both the Web-based course and the evaluation were available for 18 months.

#### Mode of Delivery, Content, and Use Parameters

Using different teaching methods, the online course provided the core characteristics of MI [24]. In total, the course comprised 49 slides. The course included information and summary boards and 3 case studies on using MI with patients suffering from chronic conditions typical for a primary care setting. The cases were presented using 2 videos showing consultation scenarios regarding overweight (one showing patient and nurse interaction and the other showing patient and physician interaction), 1 dialogue between the patient and physician on excessive alcohol consumption, and an animation on starting long-term medication in a patient suffering from type 2 diabetes and hypertension. All cases were accompanied by practical exercises.

It took about 45 min to work through the Web-based course. Users could take the course in their own time. Users could navigate within the course as desired.

#### Online Evaluation Questionnaire

Inspired by the Behaviour Change Counselling Index (BECCI) [25], a self-administered questionnaire containing 17 items was developed (see Table 1)*.* Different-level Likert scales and open-ended questions were given as answer options.

With regard to the focus of this publication, that is, how various health professionals evaluated the practicality of applying the methods they learned in the MI course, a pivotal question was about the *applicability of the skills and knowledge obtained from the course in clinical practice* (item 3). Other study-relevant items were regarding the improvement of communication skills when talking to patients about behavior change (items 7, 8, and 9). The 3 lowest answer options (not at all, minimal, to some extent) were summarized to one category, *disagree*, indicating that the participant did not concur with the particular statement.

Item 13 on the feasibility of performing MI in daily general practice and item 14 about actual plans to use MI in daily practice were summarized by mean values into one statement reflecting how participants envisage MI use in the future (answer options were coded 0=not at all, 1=minimal, 2=to some extent, 3=a certain extent, and 4=a great extent). The mean values were recoded to the standard possible responses (0=0; 0.5 to 1.5=1; 1.6 to 2.5=2; 2.6 to 3.5=3; 3.6 to 4=4). The statistically descriptive evaluation was carried out using IBM SPSS Statistics, Version 23.

#### Human Involvement, Cointerventions, and Prompts

No support or assistance was given to participants while they were taking the course or answering the questionnaire. There were no cointerventions or prompts.

## Results

### Participants

In total, 607 participants from 24 European countries evaluated the Web-based MI course. Of these, 74.0% (449/607) completed the English version and 26.0% (158/607) completed the German version. The majority of participants were GPs (355/607, 58.5%), followed by PSTs (123/607, 20.3%), medical students (84/607, 13.8%), and nurses (6/607, 1.0%). A small group did not fall within these professions (referred to as *others*; 22/607, 3.6%), and 17 out of 607 (2.8%) participants refused to specify their profession. Owing to the small number of nurses and the lack of homogeneity of the group labelled *others*, we excluded these 2 groups from the analysis. Thus, the final study sample consisted of 562 participants.

### Evaluation of Motivational Interviewing Practicality by Profession

Table 2 presents the absolute and relative frequencies of answers to the study-relevant items (3, 7, 8, 9, and the combined items 13 and 14) by profession.

### Applicability of Motivational Interviewing

Medical students were more likely to rate the applicability of the skills and knowledge (item 3) as *good* (94%, 78/84), whereas the majority of PSTs (88.6%, 109/123) rated item 3 as *excellent*. The assessment of GPs varied to a greater extent, with 51.3% (182/355) selecting *excellent*, 29.9% (106/355) selecting *good*, and 17.2% (61/355) selecting *fair*.

### Intention to Use Motivational Interviewing

When asked whether they had the intention to use MI in the future (combined items 13 and 14), 79% (67/84) of the students answered *to a certain extent*, compared with 88% (109/123) of the PSTs stating to *a great extent*. The response pattern of GPs is less definitive (Table 2).

### Improvement of Communication Skills

Regarding the improvement of communication skills, medical students agreed to *a certain extent* that after taking the course, they know better how to invite patients to talk about behavior change (85%, 72/84) or to encourage patients to talk about behavior change (81%, 68/84) and 72% (61/84) agreed to *a great extent* that they actively convey respect for the patient’s choices about behavior change.

The majority of PSTs (90.2%, 111/123) agreed to *a great extent* that they now know better how to invite patients to talk about behavior change. They also agreed to *a great extent* or *a certain extent* that they could now encourage the patient to talk about behavior change (45.5%, 56/123 and 50.4%, 62/123 respectively) or that they could convey respect for the patient (49.9%, 61/123 and 44.7%, 55/123, respectively).

GPs’ responses were more evenly distributed: 37.2% (132/355) agreed to *a great extent* and 40.6% (144/355) agreed to *a certain extent* to now knowing better how to invite patients to talk about behavior change; 36.3% (129/355) agreed *to a great extent*, 27.3% (97/355) agreed to *a certain extent*, and 33.9% (120/355) *disagreed* to encourage patients to talk about behavior change; and 40.3% (143/355) agreed *to a certain extent* to actively convey respect for the patients’ choices.

**Table 2:** Absolute and relative frequency of answers in the 3 professions: medical students, physicians in specialist training, and general practitioners.

Rating options		Medical students (N=84), n (%)	Physicians in specialist training (N=123), n (%)	General practitioners (N=355), n (%)	Total (N=562), n (%)
**Applicability of the skills and knowledge I obtained from the course in my clinical practice (Question 3)**					
	Excellent	2 (2)	109 (88.6)	182 (51.3)	293 (52.1)
	Good	79 (94)	10 (8.1)	106 (29.9)	195 (34.7)
	Fair	2 (2)	3 (2.4)	61 (17.2)	66 (11.7)
	Poor	0 (0)	1 (0.8)	0 (0.0)	1 (0.2)
	N/A^a^	1 (1)	0 (0.0)	6 (1.7)	7 (1.2)
**Motivational interviewing in daily practice is feasible and intention to use (Questions 13 and 14)**					
	Agreed to a great extent	2 (2)	109 (88.6)	137 (38.6)	248 (44.1)
	Agreed to a certain extent	67 (79)	8 (6.5)	133 (37.5)	208 (37.0)
	Disagreed	11 (13)	4 (3.3)	74 (20.9)	89 (15.8)
	N/A	4 (4)	2 (1.6)	11 (3.1)	17 (3.2)
**After taking the course I know better how to invite the patient to talk about behavior change (Question 7)**					
	Agreed to a great extent	5 (6)	111 (90.2)	132 (37.2)	248 (44.1)
	Agreed to a certain extent	72 (85)	7 (5.7)	144 (40.6)	223 (39.7)
	Disagreed	3 (3)	3 (2.4)	70 (19.7)	76 (13.5)
	N/A	4 (4)	2 (1.6)	9 (2.5)	15 (2.7)
**After taking the course I know better how to encourage the patient to talk about behavior change (Question 8)**					
	Agreed to a great extent	5 (6)	56 (45.5)	129 (36.3)	190 (33.8)
	Agreed to a certain extent	68 (81)	62 (50.4)	97 (27.3)	227 (40.4)
	Disagreed	7 (8)	3 (2.4)	120 (33.9)	130 (23.1)
	N/A	4 (4)	2 (1.6)	9 (2.5)	15 (2.7)
**After taking the course I know better how to actively convey respect for the patient’s choice about behavior change (Question 9)**					
	Agreed to a great extent	61 (72)	61 (49.6)	83 (23.4)	205 (36.5)
	Agreed to a certain extent	17 (20)	55 (44.7)	143 (40.3)	215 (38.3)
	Disagreed	2 (2)	5 (4.1)	121 (34.1)	127 (22.6)
	N/A	4 (4)	2 (1.6)	8 (2.3)	14 (2.5)

^a^N/A: not applicable.

## Discussion

### General Findings

In general, participants from 3 different levels of clinical experience positively evaluated the applicability of skills and knowledge gained by a Web-based course, and the practicality of MI in their daily routine. Our results suggested that health care professionals were able to use a Web-based training course for MI to develop skills related to health behavior change.

### Course Satisfaction and Motivational Interviewing Applicability

Nevertheless, GPs, the largest and most experienced group in our sample, were less enthusiastic about MI skills learned in the course than medical students or PSTs. Equally, GPs were more reluctant to envision MI implementation in their daily practice in the future. We have explained our results as follows: First, GPs are at the highest level of training and professional expertise in our group of participants. They may intuitively use skills similar to MI and thus the methods explored in the course might not have been entirely new to them. This may also explain why GPs did not rate the improvement of their communication skills as highly as students or PSTs. Second, it is also possible that GPs were not as pleased as their less-experienced colleagues with the Web-based format of the course. In a meta-analysis, Cook et al showed that physicians were less in favor of internet-based learning compared with alternate instructional media than medical students regarding knowledge outcome and behaviors in practice and effects on patients [18]. Third, the course instructional strategies might not have met with participants’ expectations and skills. The Dreyfus and Dreyfus model provides a framework for skill acquisition that describes developmental stages beginning with novice and progressing through advanced beginner and competent and proficient to expert [20,26]. Each of these levels is characterized by qualitative differences in knowledge, skills, attitudes, and performance [27]. The Dreyfus and Dreyfus model has been successfully adopted by medical educators to assess students and residents as they learn to practice clinical medicine [28]. Owing to differences in their knowledge level, expert and novice learners demand different instructional approaches. In fact, research indicates that instructional methods that are effective for novices either have no effect or, in some cases, depress the learning of learners with more expertise [29]. For the novice, basic science knowledge is important in providing the substrate for the analytic process of clinical reasoning. Thus, exposure of students to clinical cases and examples is important [28] and was provided by the case studies in our course. Experts, on the contrary, need challenge, ongoing experience, and exposure to interesting and complex cases to avoid complacency [28]. Thus, for GPs, our course might not have provided enough challenge. The skills and attitudes of the professionals offering MI may influence its success; they may impact on the professionals’ willingness to offer MI as well as their proficiency or effectiveness using the techniques involved [30]. The next step is to optimize the tailoring, structure, and content of a Web-based MI training course to meet participants’ specific requirements with regard to their knowledge and experience [15]. Adaptive e-learning environments (AEEs) can provide tailored instruction to health professionals and students by adapting the training to each user [31,32]. However, the effectiveness of AEEs for the education of health professionals and students of health professions remains unclear and should be investigated [32]. Future research should also explore to what extent participants of the Web-based MI course actually implemented MI in their daily routine. Factors influencing the implementation of MI might be therapeutic commitment—the motivation, task-specific self-esteem and work satisfaction toward chronic patients, and role security—the skills, knowledge, and owning a role of working with them, although this theory is debated [13,33]. Future research should focus on the causal relation between practitioners’ attitudes, their actual behavior, and care improvement strategies to enhance implementation science.

Evaluating the effects of e-learning on real clinical behavior and client outcomes still remains a challenge [34]. Zwane et al suggest that the mere fact of being surveyed might actually change behavior in medical settings [35]. However, a standard, feasible, and preferred method for establishing MI adherence in practice has not yet been developed [9]. With regard to patient benefit, Rochfort et al showed that when health professionals undergo training in empowering patients for self-management of chronic conditions, it is possible to achieve improvement in patients’ self-efficacy, autonomy and motivation to change, functional capacity, pain-free days, and quality of life [36].

### Strength and Limitations

The strength of this study was its focus on the participants of a Web-based MI course from 3 different levels of professional expertise. Thus, we were able to paint a unique and user-oriented picture of MI practicality. As limitations, we neither had information on the eligible study population nor on nonresponders. Therefore, a selection effect cannot be excluded, which may result in overestimated positive results. In a heterogeneous group such as ours, including medical professionals with very limited time resources, we opted for as few questions as possible; thus, we did not collect data on the sex or age of the participants. The BECCI survey was developed and validated as a clinical assessment. The modified version as a self-assessment could not be validated for this study because of limited resources. The questionnaire was self-reported only; we did not validate the participants’ self-reported professional level. At the time of responding to the items, respondents may not have answered truthfully, a phenomenon known as social desirability bias [37]. Gaining valid answers to questions is an age-old problem in offline and online survey research. However, we did not ask overly sensitive questions, so we assume that the answers were truthful in general. Measuring outcomes from training in enabling behavior change in patients should go beyond the simple measurement of acquisition of new skills by clinicians. It needs to show improvement in patient outcomes. In addition, the primary care setting is an environment within the health care sector which has a complex and unique set of barriers and benefits to supporting behavior change over time [38], and so we call for behavior change research to be conducted in family practice.

### Conclusions

The knowledge and skills obtained by the Web-based MI course were assessed by the participants as being beneficial and appropriate for use in primary care practice. However, participants evaluated various aspects of the course differently, depending on their level of expertise. Before we recommend this course for wider use, the actual change in the behavior of the participating clinicians and the benefit to their patients should be investigated, and data other than self-reports should be incorporated.

## Abbreviations

AEEs: adaptive e-learning environments
BECCI: Behaviour Change Counselling Index
EQuiP: European Society for Quality and Safety in Family Practice
GPs: general practitioners
MI: motivational interviewing
PSTs: physicians in specialist training
WONCA: World Organization of National Colleges, Academies and Academic Associations of General Practitioners/Family Physicians

## References

[1] Miller Wr, Rollnick S (2019). Motivational Interviewing: Helping People Change, 3rd Edition (Applications of Motivational Interviewing).

[2] Rubak S, Sandbaek A, Lauritzen T, Christensen B (2005). Motivational interviewing: a systematic review and meta-analysis. Br J Gen Pract.

[3] Miller WR, Benefield RG, Tonigan JS (1993). Enhancing motivation for change in problem drinking: a controlled comparison of two therapist styles. J Consult Clin Psychol.

[4] Lundahl B, Moleni T, Burke BL, Butters R, Tollefson D, Butler C, Rollnick S (2013). Motivational interviewing in medical care settings: a systematic review and meta-analysis of randomized controlled trials. Patient Educ Couns.

[5] Knight KM, McGowan L, Dickens C, Bundy C (2006). A systematic review of motivational interviewing in physical health care settings. Br J Health Psychol.

[6] Söderlund LL, Madson MB, Rubak S, Nilsen P (2011). A systematic review of motivational interviewing training for general health care practitioners. Patient Educ Couns.

[7] Anstiss T (2009). Motivational interviewing in primary care. J Clin Psychol Med Settings.

[8] Midboe AM, Cucciare MA, Trafton JA, Ketroser N, Chardos JF (2011). Implementing motivational interviewing in primary care: the role of provider characteristics. Transl Behav Med.

[9] Barwick M, Bennett L, Johnson S, McGowan J, Moore J (2012). Training health and mental health professionals in motivational interviewing: a systematic review. Child Youth Serv Rev.

[10] Platt L, Melendez-Torres GJ, O'Donnell A, Bradley J, Newbury-Birch D, Kaner E, Ashton C (2016). How effective are brief interventions in reducing alcohol consumption: do the setting, practitioner group and content matter? Findings from a systematic review and metaregression analysis. BMJ Open.

[11] The Health Foundation.

[12] Rollnick S, Butler CC, Kinnersley P, Gregory J, Mash B (2010). Motivational interviewing. Br Med J.

[13] Keurhorst M, Anderson P, Heinen M, Bendtsen P, Baena B, Brzózka K, Colom J, Deluca P, Drummond C, Kaner E, Kłoda K, Mierzecki A, Newbury-Birch D, Okulicz-Kozaryn K, Palacio-Vieira J, Parkinson K, Reynolds J, Ronda G, Segura L, Słodownik L, Spak F, van Steenkiste B, Wallace P, Wolstenholme A, Wojnar M, Gual A, Laurant M, Wensing M (2016). Impact of primary healthcare providers' initial role security and therapeutic commitment on implementing brief interventions in managing risky alcohol consumption: a cluster randomised factorial trial. Implement Sci.

[14] Clark Rc, Mayer Re (2019). E-learning And The Science Of Instruction: Proven Guidelines For Consumers And Designers Of Multimedia Learning.

[15] Fontaine G, Cossette S, Heppell S, Boyer L, Mailhot T, Simard M, Tanguay J (2016). Evaluation of a web-based e-Learning platform for brief motivational interviewing by nurses in cardiovascular care: a pilot study. J Med Internet Res.

[16] Admiraal W, Lockhorst D (2009). E-learning in small and medium-sized enterprises across Europe. Int Small Bus J.

[17] Fontaine J, Zheng K, Van De Ven C, Li H, Hiner J, Mitchell K, Gendler S, Hanauer DA (2016). Evaluation of a proximity card authentication system for health care settings. Int J Med Inform.

[18] Cook DA, Levinson AJ, Garside S, Dupras DM, Erwin PJ, Montori VM (2008). Internet-based learning in the health professions: a meta-analysis. J Am Med Assoc.

[19] Means B, Toyama Y, Murphy R, Bakia M (2009). US Department of Education.

[20] Dreyfus H (1988). Mind Over Machine.

[21] World Organization of Family Doctors Europe.

[22] Equip elearning.

[23] [SoSci Survey: The Online Questionnaire].

[24] Droppa M, Lee H (2014). Motivational interviewing: a journey to improve health. Nursing.

[25] Lane C (2002). The Behaviour Change Counselling Index (BECCI) - Manual for coding behaviour chance counselling.

[26] Alexander PA (2003). The development of expertise: the journey from acclimation to proficiency. Educ Res.

[27] Mohedas I, Daly S, Sienko K (2016). Use of skill acquisition theory to understand novice to expert development in design ethnography. Int J Eng Educ.

[28] Carraccio CL, Benson BJ, Nixon LJ, Derstine PL (2008). From the educational bench to the clinical bedside: translating the Dreyfus developmental model to the learning of clinical skills. Acad Med Aug.

[29] Clark R, Nguyen F, Sweller J (2006). Efficiency in Learning: Evidence–Based Guidelines to Manage Cognitive Load.

[30] Simons L, Jacobucci R, Houston H (2006). Novice, seasoned and veteran counselors' views of addiction treatment manuals: the influence of counselor characteristics on manual usefulness. J Psychoactive Drugs Dec.

[31] Brusilovsky P, Peylo C (2003). Adaptive and intelligent web-based educational systems. Int J Artif Intell Educ.

[32] Fontaine G, Cossette S, Maheu-Cadotte M, Mailhot T, Deschênes M, Mathieu-Dupuis G (2017). Effectiveness of adaptive e-learning environments on knowledge, competence, and behavior in health professionals and students: protocol for a systematic review and meta-analysis. JMIR Res Protoc.

[33] O'Donnell A, Wallace P, Kaner E (2014). From efficacy to effectiveness and beyond: what next for brief interventions in primary care?. Front Psychiatry.

[34] Sinclair PM, Kable A, Levett-Jones T, Booth D (2016). The effectiveness of internet-based e-learning on clinician behaviour and patient outcomes: A systematic review. Int J Nurs Stud.

[35] Zwane AP, Zinman J, Van Dusen E, Pariente W, Null C, Miguel E (2011). Being surveyed can change later behaviorrelated parameter estimates. Proc Natl Acad Sci.

[36] Rochfort A, Beirne S, Doran G, Patton P, Gensichen J, Kunnamo I, Smith S, Eriksson T, Collins C (2018). Does patient self-management education of primary care professionals improve patient outcomes: a systematic review. BMC Fam Pract.

[37] Demetriou C, Ozer B, Essau C, Cautin RL, Lilienfeld SO (2015). Self-report questionnaires. The Encyclopedia of Clinical Psychology.

[38] Grol R, Grimshaw J (2003). From best evidence to best practice: effective implementation of change in patients' care. Lancet.

[39] EQuiP (2012). World Organization of Family Doctors.

